# Correlation between auto/mitophagic processes and magnetic resonance imaging activity in multiple sclerosis patients

**DOI:** 10.1186/s12974-019-1526-0

**Published:** 2019-06-27

**Authors:** Massimiliano Castellazzi, Simone Patergnani, Mariapina Donadio, Carlotta Giorgi, Massimo Bonora, Enrico Fainardi, Ilaria Casetta, Enrico Granieri, Maura Pugliatti, Paolo Pinton

**Affiliations:** 1 Department of Biomedical and Specialist Surgical Sciences, Section of Neurological, Psychiatric and Psychological Sciences, University of Ferrara, Ferrara, Italy; 2 Interdepartmental Research Center for the Study of Multiple Sclerosis and Inflammatory and Degenerative Diseases of the Nervous System, University of Ferrara, Ferrara, Italy; 3 Department of Morphology, Surgery and Experimental Medicine, Section of Pathology, Oncology and Experimental Biology, Laboratory for Technologies of Advanced Therapies (LTTA), University of Ferrara, Ferrara, Italy; 4 Maria Cecilia Hospital, GVM Care and Research, Cotignola, Ravenna, 48033, Italy; 5 Department of Experimental and Clinical Biomedical Sciences, University of Florence, Florence, Italy

**Keywords:** Multiple sclerosis, Autophagy, Mitophagy, Biomarkers, Magnetic resonance imaging

## Abstract

**Background:**

An alteration of autophagy and mitophagy, two highly conserved lysosome-dependent degradation pathways involved in the maintenance of cellular homeostasis, has been associated with multiple sclerosis (MS).

**Objective:**

To search the level of autophagy-related 5 (ATG5) and Parkin proteins, as markers of autophagy and mitophagy respectively, and lactate in a cohort of MS patients.

**Methods:**

Cerebrospinal fluid (CSF) and serum samples from 60 MS patients were analyzed: 30 with magnetic resonance imaging (MRI) evidence of disease activity, gadolinium (Gd)-based contrast agent positive (Gd+), and 30 without MRI evidence of disease activity (Gd−). ATG5, Parkin, and lactate were measured using commercially available products.

**Results and conclusions:**

Serum levels of ATG5, Parkin, and lactate were more elevated in Gd+ than in Gd− MS patients (*p* < 0.0001), and CSF concentrations of ATG5 and Parkin were greater in Gd+ than in Gd− MS (*p* < 0.0001). Our results demonstrated that molecular markers of autophagy and mitophagy are increased in CSF of MS patients during the active phases of the disease and that these catabolic markers, together with lactate, are also remarkably augmented in blood suggesting a role of these processes in MS pathogenesis and the possible use of these molecules as biomarkers of disease activity.

## Introduction

Autophagy, a lysosome-dependent degradation pathway, is a highly conserved complex cellular mechanism involved in the maintenance of cellular homeostasis through the degradation of senescent subcellular organelles, infectious agents, and misfolded proteins [1].

Increasing evidence indicates that autophagy is involved in various physiological processes and in the pathogenesis of complex diseases, such as autoimmune disorders, tumors, and metabolic disorders [2]. In the immune system, autophagy can act on four main levels: (i) in the removal of intracellular pathogens [3], (ii) in the development of T and B lymphocytes [4, 5], (iii) in the pro-inflammatory signal cascade [6], and (iv) in the secretory pathway [7]. Based on its functions within the immune system, autophagy may play a pathogenic and/or therapeutic role in autoimmune diseases. Previous studies have shown an implication of autophagy in systemic lupus erythematosus, psoriasis, rheumatoid arthritis, inflammatory bowel disease, and MS [2]. In confirmation of this, immunohistochemical analysis has unveiled the presence of autophagic features in MS brain tissue samples [8]. Furthermore, a gain of expression of autophagic markers was found in experimental autoimmune encephalomyelitis (EAE) animal models [9] and conditional knockdown of genes related to autophagy has shown therapeutic effects in the animal model of MS [10].

A selective form of autophagy is also involved in the elimination of damaged mitochondria in a process called mitophagy [11]. Suppression of mitophagy leads to accumulation of aberrant mitochondria, reactive oxygen species (ROS) overload, and consequent mitochondrial malfunction [12].

In the immune system, proper mitochondrial function is a fundamental prerequisite for inflammatory responses and host defense [13, 14]. Accordingly, impairments in the correct functioning of the mitochondrial population can lead to activation of inflammatory signaling pathways [15] with the consequent establishment of a chronic inflammatory condition that could result in the development of autoimmune diseases [16].

Mitochondrial dysfunctions are also implicated in normal and physiological aging processes as well as in a broad spectrum of age-related disorders, including some neurodegenerative diseases such as Parkinson’s and Alzheimer’s [17]. Regarding the MS, it is possible to list specific hallmarks of mitochondrial anomalies during the development and the progression of the disease. Oxidative damage and anomalous mitochondrial protein functions are the most common cause of mitochondrial dysfunction found in MS [18]. Several studies showed that mtDNA mutations and cell death mechanisms, like apoptosis and necrosis, are involved during the pathophysiology of MS [19]. Recently, we also demonstrated that mitochondrial impairments observed in MS might be caused by alteration of cellular clearing mechanism. Indeed, in MS-like inflammatory conditions, we found a sustained alteration of mitochondrial functioning accompanied by adenosine monophosphate-activated protein kinase (AMPK) activation [20]. Of note, AMPK is the main positive regulator of the autophagic machinery [1]. Most importantly, we proved that molecular markers of autophagy and mitophagy were elevated in the central nervous system (CNS) of MS patients compared to neurological controls, suggesting a role of these two mechanisms in the pathogenesis and/or development of MS [21]. Moreover, in the same work, we found that with respect to other neurological diseases and healthy individuals, autophagy- and mitophagy-related molecules were also elevated at the systemic level in MS patients, suggesting a potential use of these molecules as a diagnostic/prognostic biomarker in the course of MS.

The present study aimed to investigate the potential role of ATG-5 and Parkin proteins, as biomarkers of autophagy and mitophagy, respectively, in a cohort of MS patients stratified according to MRI activity. Moreover, in the same population, serum levels of lactate were measured as an indicator of mitochondrial dysfunction [22].

## Materials and methods

### Study design

This study retrospectively included 60 patients with relapsing-remitting MS according to the currently accepted criteria [23]. At the time of sample collection, (a) disease severity was scored using Kurtzke’s Expanded Disability Status Scale (EDSS) [24] and (b) the presence of relapse was recorded as a clinical activity. Lumbar puncture was performed as part of the diagnostic work-up for a suspect of MS, and subsequently, all patients were “naïve” for disease-modifying treatment. Moreover, none of the study subjects underwent treatment with immunosuppressants or immune-modulating drugs, including steroids, during the period of 6 months before the study. The study was approved by the Committee for Medical Ethics in Research of Ferrara, and written consent to study participation was obtained from all subjects.

### Sample handling

Paired CSF and serum samples were collected from MS patients for purposes of diagnosis and measured under the same conditions. CSF samples were obtained through atraumatic lumbar puncture. CSF cell count was performed on untreated samples within 2 h from the withdrawal, and the threshold of 4 white blood cells (WBC)/μl was used to indicate a “normal cell count” [25]. Cell-free CSF and serum were obtained after centrifugation at 3000*g* at 20 °C for 15 min. Supernatants were collected, under sterile conditions in aliquots of 500 μl, coded, frozen, and stored at − 80 °C until assay. Serum and CSF albumin levels of were determined by immunochemical nephelometry with the Beckman Immage 800 system (Beckman Instruments, Fullerton, CA, USA), and the blood-brain barrier permeability was measured through the calculation of the CSF/serum albumin quotient (QAlb) [25]. Intrathecal immunoglobulin synthesis was investigated through isoelectric focusing followed by IgG-specific immunoblotting in paired CSF and serum samples [26].

### Magnetic resonance imaging analysis

**Fig. 1:**
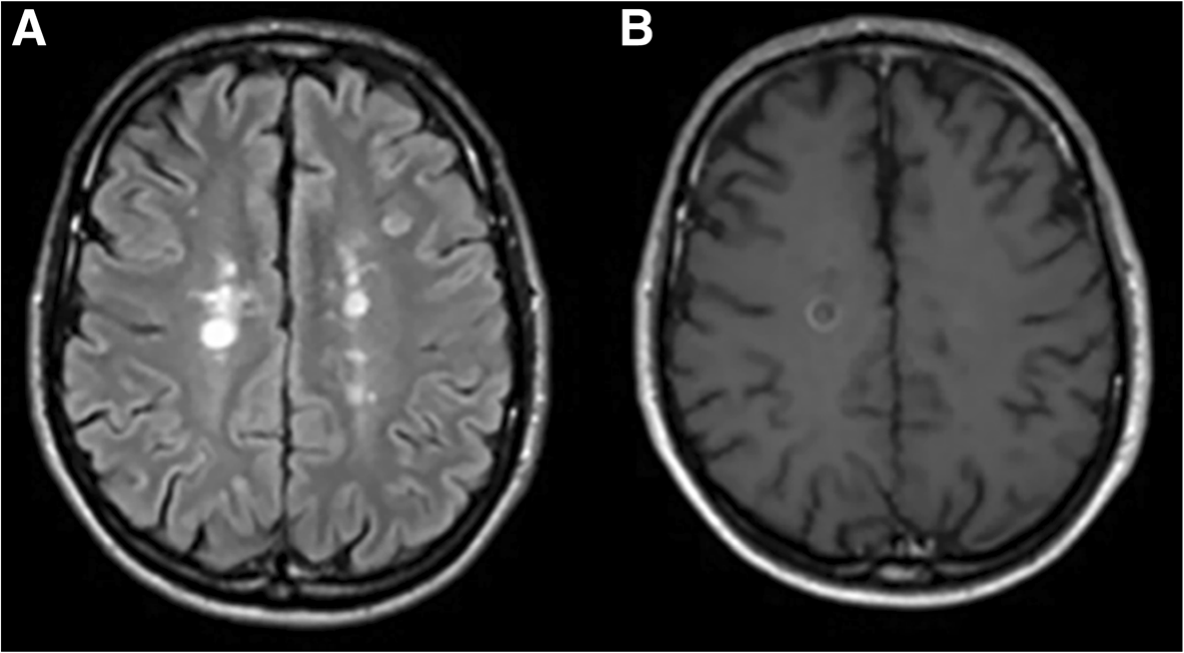
Magnetic resonance imaging (MRI) scan in a 35-year-old woman with relapsing-remitting multiple sclerosis (MS) imaged at 36 h after lumbar puncture showing bilateral multiple and partially confluent hyperintense lesions in periventricular white matter on fluid-attenuated inversion recovery (FLAIR) axial images (**a**). The ring enhancement characterizing the lesion located in the left hemisphere, at the level of centrum semiovale, was recognized on contrast-enhanced T1-weighted axial spin echo images (**b**) by one neuroradiologist, but not by the other. After consensus, this lesion was classified as Gadolium (Gd) enhanced and the patient as MRI active (Gd+). The consensus was needed for the same reasons in 5/60 (8.3%) patients included in the study

All MS patients underwent brain MRI scans within 48 h from lumbar puncture on a 1.5-Tesla MRI unit (GE Signa Horizon, General Electric Medical Systems, Milwaukee, WI, USA). Routinely used T1-weighted axial spin echo images were obtained approximately 10 min after intravenous injection of 0.1 mmol/kg of gadopentetic acid (Gd-DTPA) in each patient. Lesions showing Gd enhancement on T1-weighted scans were defined as indicative of MRI activity, since they occur only at sites of active inflammation, are considered a powerful tool to detect disease activity in MS, and are more sensitive in measuring disease activity than clinical examination [27]. Accordingly, MS patients with one or more Gd-enhancing lesions (Gd+) were classified as MRI active and MS patients without Gd-enhancing lesions (Gd-) were classified as MRI inactive. Two neuroradiologists independently reviewed all neuroimaging data (with 15 and 20 years experience, respectively) blinded to the patients’ clinical and laboratory data. Discrepancies between readers were resolved by consensus adjudication (Fig. 1).

### ATG-5 and Parkin level determination

According to our previous study [21], CSF and serum levels of ATG-5 and Parkin were determined using two commercially available ELISA kits (My Biosource, San Diego, California, USA; codes MS7209535 and MBS732278, respectively) following the instructions of the manufacturer.

### Lactate level determination

After collection, serum samples were immediately partitioned into sterile cryovials tubes and transported at − 80 °C. To prevent detrimental of serum components, repeated freeze-thaw cycles were avoided. Serum levels of lactate were determined using a colorimetric L-Lactate Assay Kit according to the manufacturer’s protocol (L-Lactate Assay Kit Colorimetric, Abcam, ab65331). Briefly, 10 μl of serum sample plus 40 μl of Lactate Assay Buffer was added to 96-plate wells with a reaction mix composed of Lactate Assay Buffer, Lactate Substrate Mix, and Lactate Enzyme Mix. After incubation for 30′, the OD at 450 nm was measured on a microplate reader. Each sample was analyzed in triplicate.

### Statistical analysis

The categorical variables were expressed in frequencies (percentages) and compared with the mean chi-square tests. The distribution of continuous variables was analyzed with the Kolmogorov-Smirnov test. (i) In the case of normal distribution of the variables, the values were presented as mean and standard deviations, the comparison between two groups by means of the student test, and the correlation between two variables was evaluated using the Pearson index. (ii) In the case of non-normal data distribution, a non-parametric statistic was used: data were presented as median and interquartile range, the comparison between two groups by the Mann-Whitney test, and the correlation between two variables was evaluated using the Spearman index.

Two-tailed tests were calculated, and a *p* value of less than 0.05 was considered significant. The SPSS® statistical package for Windows and OSX (SPSS Inc., IBM®, Somers, NY, USA) and Prism® (GraphPad Software Inc.) were used for statistical analysis.

## Results

The study was conducted on 60 MS individuals: 30 with MRI evidence of disease activity (Gd+) and 30, age and sex matched, without MRI evidence of disease activity (Gd−). The main clinical-demographic features of the study population are reported in Table 1.

**Table 1:** Clinical and demographic main features of multiple sclerosis (MS) study population grouped according to magnetic resonance imaging (MRI) evidence of disease activity

	Gd− MS (*n* = 30)	Gd+ MS (*n* = 30)
Sex (F), *N* (%)	21 (70.0)	21 (70.0)
Mean (SD) age (years) at study time	45.3 (11.9)	42.8 (11.3)
Median (IQR) EDSS at study time	2.3 (1.0–3.4)	1.8 (1.0–2.8)
Clinical activity at study time, *N* (%)	21/30 (70)	26/30 (86.7)
CSF characteristics		
Median (IQR) QAlb	4.5 (3.3–5.8)	4.7 (3.9–5.9)
Normal cell count (< 4 WBC/μl), *N* (%)	29 (97)	24 (80)
CSF-restricted IgG OCB (pos), *N* (%)	22 (73.3)	23 (76.7)

*CSF* cerebrospinal fluid, *EDSS* expanded disability status scale, *Gd+* magnetic resonance imaging (MRI) evidence of disease activity, *Gd−* no MRI evidence of disease activity, *IQR*, interquartile range, *MS* multiple sclerosis, *OCB* oligoclonal bands, *QAlb* CSF/serum albumin quotient, *SD* standard deviation, *WBC* white blood cells

### Serum and cerebrospinal fluid levels of ATG-5

**Fig. 2:**
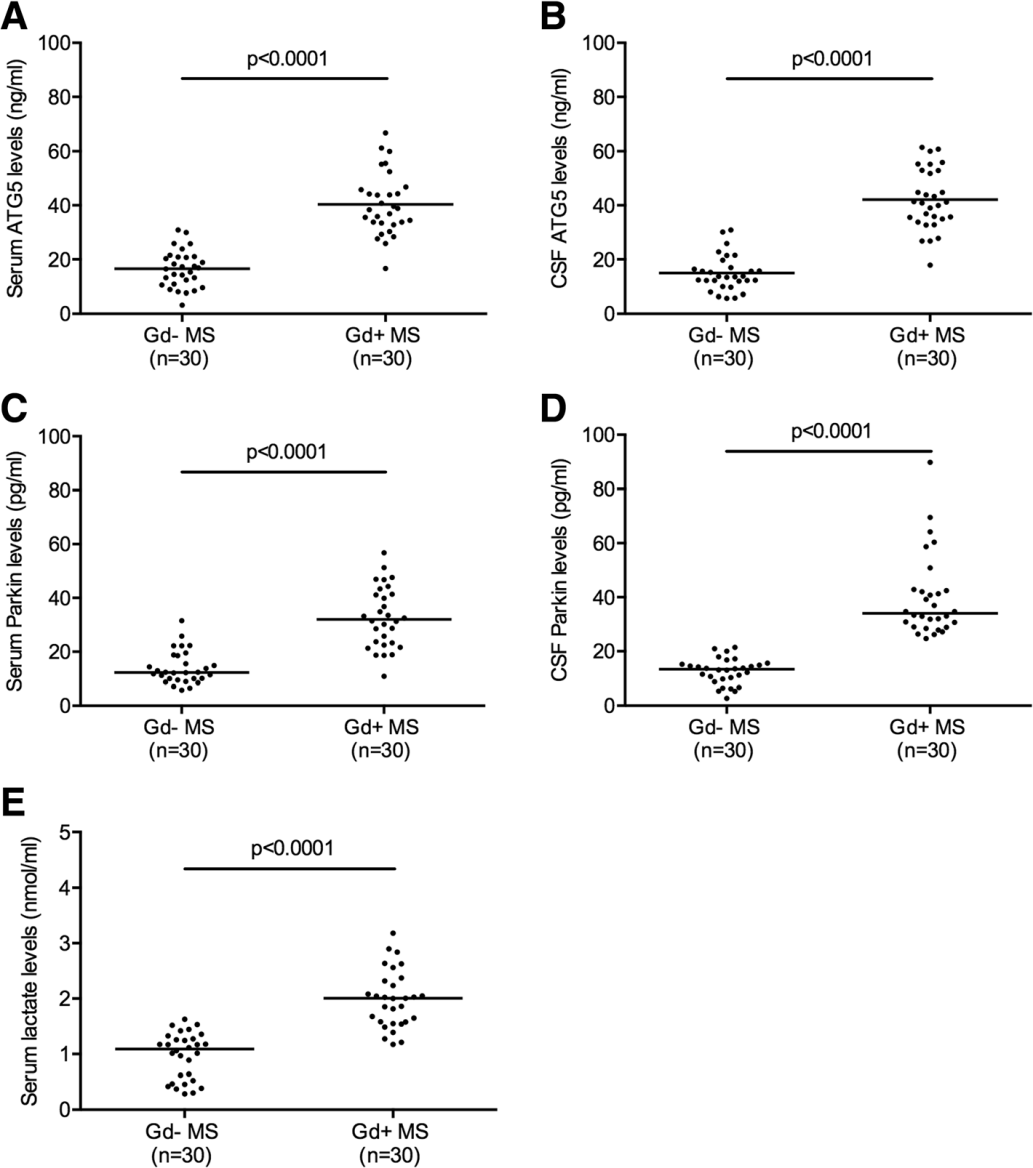
Autophagy-related 5 (ATG5), Parkin, and lactate serum and cerebrospinal fluid (CSF) levels in 30 multiple sclerosis (MS) patients with magnetic resonance imaging (MRI) evidence of disease activity (Gd+) and 30 MS patients without MRI evidence of disease activity (Gd−). ATG5 serum and CSF levels were higher in Gd+ MS patients than in Gd− (unpaired t test; *p* < 0.0001) (**a**, **b**). Parkin serum and CSF concentrations were increased in Gd+ MS patients than in Gd− (Mann-Whitney; *p* < 0.0001) (**c**, **d**). Lactate serum levels were more elevated in Gd+ MS than in Gd− (Mann-Whitney; *p* < 0.0001) (**e**). Each point represents a single observation. Horizontal bars indicate means (**a**, **b**) or medians (**c**–**e**)

Serum concentrations of ATG-5 were more elevated in Gd+ MS (mean ± standard deviation (SD) 40.41 ± 11.49 ng/ml) than in Gd− MS patients (mean ± SD 16.53 ± 6.80 ng/ml) (unpaired t test; *p* < 0.0001) (Fig. 2a). CSF levels of ATG-5 were increased in Gd+ MS (mean ± SD 42.10 ± 11.22 ng/ml) with respect to Gd− MS subjects (mean ± SD 14.93 ± 6.54 ng/ml) (unpaired t test; *p* < 0.0001) (Fig. 2b).

### Serum and cerebrospinal fluid levels of Parkin

Serum concentrations of Parkin were higher in Gd+ MS (median, interquartile range (IQR) 32.03, 23.10–41.94 pg/ml) than in Gd− MS patients (median, IQR 12.29, 9.90–18.61 pg/ml) (Mann-Whitney; *p* < 0.0001) (Fig. 2c). CSF levels of Parkin were greater in Gd+ MS (median, IQR 34.08, 29.02–42.56 pg/ml) than in Gd− MS subjects (median, IQR 11.34, 9.55–15.32 pg/ml) (Mann-Whitney; *p* < 0.0001) (Fig. 2d).

### Serum levels of lactate

Serum concentrations of lactate were more elevated in Gd+ MS (median, IQR 2.01, 1.57–2.33 nmol/ml) than in Gd− MS patients (median, IQR 1.09, 0.51–1.29 nmol/ml) (Mann-Whitney; *p* < 0.0001) (Fig. 2e).

Serum levels of lactate were positively correlated to ATG-5 and Parkin in MS patients analyzed as a whole (both Pearson; *p* < 0.0001), while no statistical significant correlations were found by grouping MS patients according to MRI evidence of disease activity (Table 2).

**Table 2:** Comparison between serum levels of lactate and autophagy-related 5 (ATG-5) and Parkin in multiple sclerosis (MS) patients analyzed as a whole and grouped according to magnetic resonance imaging (MRI) evidence of disease activity

		Serum lactate levels (nmol/ml)
MS (*n* = 60)		
Serum ATG-5 levels (pg/ml)		*r* = 0.5897; *p* < 0.0001 (Pearson)
Serum Parkin levels (ng/ml)		*r* = 0.4855; *p* < 0.0001 (Pearson)
Gd− MS (*n* = 30)		
Serum ATG-5 levels (pg/ml)		*r* = 0.1213; *p* = 5233 (Spearman)
Serum Parkin levels (ng/ml)		*r* = − 0.3246; *p* = 0.0801 (Spearman)
Gd+ MS (*n* = 30)		
Serum ATG-5 levels (pg/ml)		*r* = − 0.01697; *p* = 0.9291 (Spearman)
Serum Parkin levels (ng/ml)		*r* = − 0.08174; *p* = 0.6565 (Spearman)

*Gd+* MRI evidence of disease activity, *Gd−* no MRI evidence of disease activity

## Discussion

In the present study, we demonstrated that molecular markers of autophagy and mitophagy are increased in the CNS of MS patients during the active phases of the disease. Furthermore, our data indicated that concentrations of these catabolic markers, together with lactate (a reliable indicator of mitochondrial malfunctioning), are remarkably augmented in peripheral blood of the same patients.

Our results provide new information on these biological markers that we had previously shown to be increased, within the CNS and at the systemic level, in patients with MS compared to other neurological disorders and healthy individuals [21].

At today, the role of autophagy in autoimmune diseases and in particular in MS is still debated. Autophagy exerts a complex function in cell life, acting either as a pro-survival or as a cell death mechanism. ATG5-deficient CD4+ and CD8+ T cells have a normal growth in the thymus, but fail to repopulate the periphery due to massive cell death and fail to proliferate after stimulation efficiently [4]. On the other hand, T regulatory (Treg) cell-specific deletion of ATG5 and ATG7 resulted in a reduction in frequencies and survival of these cells causing a defective self-tolerance [28]. These data depict a critical role of autophagy in the development and function of lymphocytes by suggesting that this cellular mechanism may be essential for (i) survival, (ii) proliferation, and (iii) activation of T lymphocytes. Our results strongly confirm that an increase of ATG5 could be associated with demyelination as it was described in MS lesions and the animal model of MS, the EAE [9]. Similarly, increases in ATG5 levels (mRNA and protein amount) were also found in peripheral blood mononuclear cell (PBMC) obtained from MS patients who are treatment naïve [29]. In this work, the authors also found a direct correlation among ATG5 and pro-inflammatory cytokines levels, suggesting a strong relationship among ATG5 and inflammation, but they did not observe any change in the expression of other autophagy genes. In contrast to this, significant variations in the expression of several autophagic regulators were recognized in a different and larger population which comprised treated and untreated MS patients [30]. Future studies investigating the role of MS therapeutic protocols may help to solve this issue.

A particular form of autophagy is called mitophagy: a mechanism by which damaged or dysfunctional mitochondria are addressed to lysosomal degradation. Accumulation of damaged mitochondria can lead an excessive ROS production, elevated cytoplasmic calcium levels, and mitochondrial DNA release, which may activate immune signaling pathways with the final consequence of causing a release of inflammatory cytokines [13, 31, 32]. Furthermore, the release of these cytokines may subsequently promote the release of other soluble inflammation mediators including IL-23 and IL-17 [33]. All these data indicate that in addition to being essential for normal cellular homeostasis, mitophagy may also represent a crucial mechanism in regulating and controlling inflammatory responses.

Recently, we described an increase of mitophagic-related protein, Parkin, in CSF and serum of MS patients compared to other subjects affected by inflammatory and non-inflammatory CNS diseases as well as to healthy donors (only in serum). Parkin is a ubiquitin ligase, with an amino-terminal ubiquitin-like domain and a carboxyl-terminal ubiquitin ligase domain [34], that is recruited from the cytosol to depolarized mitochondria to promote the selective removal of the damaged organelle [35]. The presence of augmented levels of Parkin in CSF levels was recently confirmed by Kristofikova and colleagues, who found increased levels of the kinase bound to the mitochondrial protein 17β-hydroxysteroid dehydrogenase type 10 (17β-HSD10) in CSF of MS-affected patients [36]. Interestingly, no difference was found when 17β-HSD10 was dissociated with Parkin, confirming the importance of the kinase during MS. Moreover, according to the results of that paper, CSF levels of 17β-HSD10 were not considered a valuable biomarker for the early diagnosis or for the progression of MS.

Mitophagy impairment is associated with aging and to a wide spectrum of age-related diseases, including neurodegenerative disorders such as Parkinson’s disease and Alzheimer’s disease [17].

Our previous data indicated that an increase in mitophagic activity was associated to MS, and the present results showed for the first time that this increase is associated explicitly to the active phase of the disease as demonstrated by the presence of contrast-enhancing lesions at the MRI analysis.

Recently, serum lactate has been indicated as a potential biomarker of mitochondrial dysfunction in MS patients and that this molecule is associated with the progression of the disease and to the disability accumulation [22].

Our data add further information on the role of lactate in MS subjects. In fact, in our population, serum levels of lactate, together with the autophagy- and mitophagy-related molecules, were increased in MS patients with gadolinium contrast enhancement, suggesting that these metabolic imbalances are more pronounced during the active phase of the disease. These findings corroborated with studies showing that during differentiation events, oligodendrocyte cells consumed lactate to ameliorate hypomyelination induced by low energetic conditions [37, 38]. Similar observations were also found in maturating oligodendrocytes exposed to inflammatory conditions, where increased lactate levels were accompanied by a progressive loss of mitochondrial functioning [20]. Considering all these aspects, it is possible to hypothesize that during the active state of the pathology, the auto/mitophagic events are activated by loss of the proper mitochondrial functions as denoted by increased lactate levels. Similarly, increases in ATG5 levels (mRNA and proteic amount) were also found in PBMC obtained from treatment-naïve MS patients [29]. In that work, authors also found a direct correlation among ATG5 and pro-inflammatory cytokines levels, suggesting a strong relationship among ATG5 and inflammation, but not observed any change in the expression of other autophagy genes. In contrast to this, significant variations in the expression of several autophagic regulators were recognized in a different and larger population which comprised treated and untreated MS patients [30]. Future studies investigating the role of MS therapeutic protocols may help to solve this issue.

Overall, our results started to bring new lights on the role of autophagy and mitophagy in CNS pathology and in particular in MS. If on the one hand it is accepted that the reduction of autophagy and mitophagy processes within the CNS is mainly related to neurodegenerative diseases, such as Alzheimer’s disease and Parkinson’s disease [17], on the other hand, our studies show that an increase of soluble markers of these two metabolic processes is related to the presence of a chronic CNS inflammation and that these markers further increase during the acute phase of the inflammatory reaction. Moreover, the serum increase we observed of autophagy, mitophagy, and mitochondrial malfunctions biomarkers seems to indicate that these metabolic processes are correlated to the immune response that starts in the periphery and enters the CNS in an “outside-in” model of autoimmune reaction. Most importantly, the presence of these molecules in blood, an easier accessible biological fluid, and their correlation with the disease activity together suggest a potential role as markers of the natural history of the disease that correlates longitudinally with known clinical indices, following the definition of “type 0 biomarker” [39].

## Conclusions

Our study indicates that in the CNS of MS patients, autophagic and mitophagic markers are increased during the active phase of the disease as a consequence of the inflammatory reaction that occurs within the brain. In parallel, we denoted that these molecules, together with lactate, are also increased in peripheral blood, probably reflecting an immune activation taking place in periphery. The presence of these metabolic markers not only in CSF but also in an easily accessible body fluid like blood suggests a potential role of these molecules as biomarkers for disease activity and progression in course of MS. Thus, as was the case for other biomarkers [40], further studies are warranted to validate the use of these promising molecules, in particular (i) on patients presenting clinically isolated syndromes and on healthy controls to evaluate the capability to guide an early diagnosis of MS, and (ii) during immunomodulating treatment for the monitoring of disease activity and progression.

## Data Availability

The datasets analyzed during the current study is available from the corresponding author on reasonable request.

## Abbreviations

17β-HSD10: 17β-Hydroxysteroid dehydrogenase type 10
AMPK: Adenosine monophosphate-activated protein kinase
ATG5: Autophagy-related 5
CNS: Central nervous system
CSF: Cerebrospinal fluid
EAE: Experimental autoimmune encephalomyelitis
EDSS: Expanded Disability Status Scale
Gd: Gadolinium
Gd+: Gadolinium-based contrast agent positive
Gd−: Gadolinium-based contrast agent negative
Gd-DTPA: Gadopentetic acid
MRI: Magnetic resonance imaging
MS: Multiple sclerosis
PBMC: Peripheral blood mononuclear cell
QAlb: Serum albumin quotient
ROS: Reactive oxygen species
Treg: T regulatory
WBC: White blood cells
